# Macrophage Anti-inflammatory Behaviour in a Multiphase Model of Atherosclerotic Plaque Development

**DOI:** 10.1007/s11538-023-01142-7

**Published:** 2023-03-29

**Authors:** Ishraq U. Ahmed, Helen M. Byrne, Mary R. Myerscough

**Affiliations:** 1 School of Mathematics and Statistics, University of Sydney, Sydney, Australia; 2 Wolfson Centre for Mathematical Biology, Mathematical Institute, University of Oxford, Oxford, UK

## Abstract

Atherosclerosis is an inflammatory disease characterised by the formation of plaques, which are deposits of lipids and cholesterol-laden macrophages that form in the artery wall. The inflammation is often non-resolving, due in large part to changes in normal macrophage anti-inflammatory behaviour that are induced by the toxic plaque microenvironment. These changes include higher death rates, defective efferocytic uptake of dead cells, and reduced rates of emigration. We develop a free boundary multiphase model for early atherosclerotic plaques, and we use it to investigate the effects of impaired macrophage anti-inflammatory behaviour on plaque structure and growth. We find that high rates of cell death relative to efferocytic uptake results in a plaque populated mostly by dead cells. We also find that emigration can potentially slow or halt plaque growth by allowing material to exit the plaque, but this is contingent on the availability of live macrophage foam cells in the deep plaque. Finally, we introduce an additional bead species to model macrophage tagging via microspheres, and we use the extended model to explore how high rates of cell death and low rates of efferocytosis and emigration prevent the clearance of macrophages from the plaque.

## Introduction

Atherosclerosis is a chronic inflammatory disorder characterised by the retention of lipoproteins and cholesterol-laden macrophages in the artery wall (Libby et al 2019; Tabas et al 2015). Certain macrophage behaviours are vital in enabling inflammation to resolve in both plaques and other inflammatory contexts. These behaviours include the emigration of macrophages from the site of inflammation and the clearance of dead cells (Tabas 2010; Llodrá et al 2004). Macrophages in advanced plaques, however, display an impaired ability to carry out normal anti-inflammatory functions (Tabas 2010; Randolph 2008). Understanding how these impaired behaviours may promote macrophage retention in early plaques is therefore important to identify the conditions that lead to advanced plaque development. In this paper, we develop a partial differential equation model for an atherosclerotic plaque based on a multiphase framework, and we use this to investigate how macrophage emigration and dead cell clearance influence the growth and composition of the early plaque.

Atherosclerotic lesions are initiated when cholesterol-carrying low-density lipoproteins (LDL) from the bloodstream are deposited in the intima (Libby et al 2019; Falk 2006). The intima is an initially thin layer that separates the endothelium (a monolayer of endothelial cells lining the interior of the artery) from the media (a thicker layer comprising muscle cells and collagen). The endothelium becomes more permeable to particles such as LDL at sites of endothelial dysfunction, which occur due to disrupted blood shear flow or endothelial cell damage (Gimbrone Jr and García-Cardeña 2013). LDL particles that have penetrated the endothelium and accumulated in the intima undergo oxidation and other forms of chemical modification to produce modified LDL (modLDL) (Madamanchi et al 2005; Yoshida and Kisugi 2010). Intimal modLDL is a potent immune trigger and stimulates the expression of adhesion molecules by endothelial cells. These adhesion molecules bind to and capture monocytes circulating in the bloodstream, which then transmigrate through the endothelium into the intima (Blankenberg et al 2003; Bobryshev 2006). Once in the intima, monocytes differentiate into macrophages, which then take up modLDL via phagocytosis (Bobryshev 2006; Moore et al 2013). The resulting cholesterol-engorged macrophages are referred to as macrophage foam cells. Early atherosclerotic lesions consist largely of modLDL, macrophage foam cells, and debris from dead cells.

In atherosclerosis and other forms of inflammation, macrophages undergo a form of programmed cell death called apoptosis (Cohen and Mosser 2013; Van Vré et al 2012). Apoptosis is normal even in healthy tissue, and apoptotic cells express find-me and eat-me signals to encourage their clearance by live macrophages via efferocytosis. In chronic inflammatory conditions such as advanced atherosclerosis, the accumulation of apoptotic material will often overwhelm macrophages’ efferocytic capacity. This is especially likely in cases where rates of macrophage death are elevated due to high levels of ingested cytotoxic material (such as cholesterol), or where efferocytosis itself becomes defective. Both of these are observed in atherosclerotic plaques (Yurdagul Jr et al 2018; Schrijvers et al 2005). Uncleared apoptotic cells will eventually undergo an uncontrolled form of cell death called necrosis. Necrotic macrophages are highly problematic due to their lower production of “find-me” and “eat-me” signals. Necrotic material is also highly inflammatory and will attract more macrophages which are themselves likely to undergo necrosis, thereby perpetuating the cycle. A major characteristic of advanced plaques is the presence of a large necrotic core, consisting of lipids and debris released by necrotic cells (Sakakura et al 2013). The inefficient efferocytic clearance of apoptotic material is therefore an important precursor to the development of a vulnerable plaque state that is more likely to rupture.

The emigration of macrophages from sites of inflammation is a critical part of inflammation resolution in other inflammatory conditions (Lawrence and Gilroy 2007; Bellingan et al 1996) and is believed to play an important part in atherosclerosis as well, although its mechanisms are not yet well understood. Experimental studies have measured the expression of receptors such as CCR7 that are known to be involved in macrophage emigration in other inflammatory conditions and find that higher expression of these receptors is correlated with reduced plaque size (Trogan et al 2002; Finney et al 2017). Several studies suggest that emigration most likely happens via migration into lymphatic vessels that connect to the outer artery. One such study found that after plaque regression was induced, macrophages that were originally present in the plaque were found in the lymph nodes (Llodrá et al 2004). Another study involving fluorescent bead-tagged macrophages found that the distance between tagged macrophages and the elastic lamina (a membrane separating the intima from the outer muscle cell layers) decreased during plaque regression, suggesting that macrophages are exiting through the outer artery instead of transmigrating across the endothelium (Williams et al 2018).

Mathematical modelling of the inflammatory response during atherosclerosis is an area of growing interest (Parton et al 2016). Much of this work uses a continuum approach to investigate the activity of monocytes and macrophages in the vessel wall and their interactions with lipoproteins. Previous work in our group has considered the accumulation and efferocytic clearance of apoptotic cells using both ODE models (Lui and Myerscough 2021) and non-spatial lipid-structured PDE models for macrophage populations in plaques (Ford et al 2019; Chambers et al 2022). An alternative model by Fok (2012) considers macrophages and dead cells in a reaction-diffusion model for plaque development. No other spatial models exist to date that explicitly consider both cell death and efferocytosis.

Most earlier spatial models model the intima using a spatial domain that remains fixed in size. More recent models employ a free boundary to allow the artery wall to expand as the plaque grows. Models by Fok (2016) and Thon et al (2018a) model the artery wall as elastic media, which can expand in response to increasing stresses caused by the accumulation of new material. Other models (Friedman and Hao 2015; Zhao and Hu 2022) employ a multiphase framework to capture the effects of cell crowding. However, none of these models incorporate macrophage emigration in a way that respects local mass conservation, although some fixed domain models incorporate an unbalanced sink term for macrophage removal due to death or emigration (Fok 2012).

In this paper, we present a free boundary multiphase model for early plaque growth that focuses on the interplay between foam cell death, efferocytosis, and emigration. We formulate the model in Sect. 2, employing a similar multiphase approach to Friedman (2015); Ward and King (1997). Our model explicitly includes cell death and the efferocytic uptake of dead cells. The model also includes macrophage emigration, which is modelled using a boundary flux rather than an unbalanced sink term. In Sect. 3, we investigate how the relative rates of foam cell death and efferocytic uptake influence plaque composition. In Sect. 4, we show how macrophage emigration can slow or halt plaque growth, and how its effectiveness depends on the availability of live foam cells in the deep plaque. In Sect. 5, we extend the model to include macrophage tagging, and we use the extended model to investigate macrophage retention in plaques. We conclude in Sect. 6 with a discussion of the results in the context of existing plaque models and biomedical studies and some suggestions for future modelling work.

## Model Formulation

**Fig. 1:**
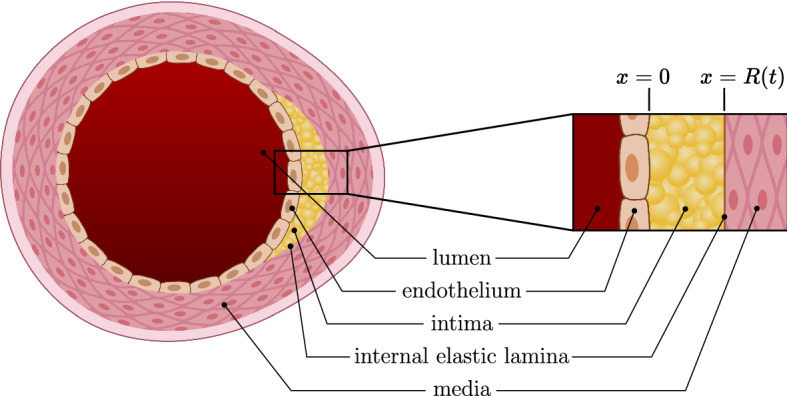
A 2-dimensional cross section of an artery, with a 1-dimensional radial cross section taken through the plaque for the problem domain

In this section, we present a general model that describes the growth of an early-stage atherosclerotic plaque. We model the intima as a 1-dimensional domain on the interval $[0,\tilde{R}(\tilde{t})]$, which represents a radial cross section through the artery wall. The $\tilde{x} = 0$ boundary corresponds to the endothelium, and the $\tilde{x} = \tilde{R}(\tilde{t})$ boundary corresponds to the elastic lamina that separates the intima from the media (see Fig. 1). We allow the $\tilde{x} = \tilde{R}(\tilde{t})$ boundary to vary with time since early plaques cause the artery to undergo compensatory enlargement. During compensatory enlargement, the artery expands outwards in a way that preserves lumenal area (Korshunov et al 2007). We also assume that the diameter of the artery is much larger than the width of the intima in early-stage plaques (Bonithon-Kopp et al 1996), so that Cartesian coordinates are a good approximation to radial coordinates. We notate all quantities with tildes for the full dimensional model and then remove tildes for the nondimensional model in Sect. 2.1.

We assume that the plaque comprises three phases, each measured in cells or equivalent cell volumes per unit length. These phases are macrophage foam cells $\tilde{f}(\tilde{x},\tilde{t})$, modified LDL (or modLDL) $\tilde{l}(\tilde{x},\tilde{t})$, and dead cellular material $\tilde{c}(\tilde{x},\tilde{t})$. For simplicity, we let $\tilde{f}(\tilde{x},\tilde{t})$ encompass all monocyte-derived cells, including M1 and M2 macrophages and dendritic cells for instance (Murray and Wynn 2011), ignoring differences in phenotypic expression. Similarly, we ignore the distinction between the various forms of chemically modified LDL, treating them as a single modLDL species $\tilde{l}(\tilde{x},\tilde{t})$. For cell death, we ignore the distinction between apoptosis and necrosis and use a generic cell death term to encompass both processes.

The phases obey the following continuity equations:

1 $$\begin{aligned} \frac{\partial \tilde{f}}{\partial \tilde{t}}&= - \frac{\partial }{\partial \tilde{x}} ( \tilde{J}_f + \tilde{v} \tilde{f} ) + \tilde{s}_f \,, \end{aligned}$$

2 $$\begin{aligned} \frac{\partial \tilde{l}}{\partial \tilde{t}}&= - \frac{\partial }{\partial \tilde{x}} ( \tilde{J}_l + \tilde{v} \tilde{l} ) + \tilde{s}_l \,, \end{aligned}$$

3 $$\begin{aligned} \frac{\partial \tilde{c}}{\partial \tilde{t}}&= - \frac{\partial }{\partial \tilde{x}} ( \tilde{J}_c + \tilde{v} \tilde{c} ) + \tilde{s}_c \,. \end{aligned}$$

In the above, $\tilde{J}_u$ and $\tilde{s}_u$ denote phase-specific flux and source terms, respectively, for the phases $u = f,\,l,\,c$, and $\tilde{v}$ is a common mixture velocity with which all phases are advected. We also assume there are no voids throughout the plaque, so that the phases obey a no-voids condition

4 $$\begin{aligned} \tilde{f} + \tilde{l} + \tilde{c} = N_0 \end{aligned}$$

everywhere, where $N_0$ denotes the total phase density, which is constant with time and space.

Foam cells internalise modLDL via phagocytosis, and dead material via efferocytosis. We model both processes with a simple mass-action term. Foam cells also undergo cell death, which we model using a linear death term. This gives the following expressions for the source terms in (1)–(3):

5 $$\begin{aligned} \tilde{s}_f&= + \tilde{\mu }_p \tilde{f} \tilde{l} + \tilde{\mu }_e \tilde{f} \tilde{c} - \tilde{\mu }_a \tilde{f} \,, \end{aligned}$$

6 $$\begin{aligned} \tilde{s}_l&= - \tilde{\mu }_p \tilde{f} \tilde{l} , \end{aligned}$$

7 $$\begin{aligned} \tilde{s}_c&= - \tilde{\mu }_e \tilde{f} \tilde{c} + \tilde{\mu }_a \tilde{f} \,, \end{aligned}$$

where $\tilde{\mu }_a$ is the macrophage foam cell death rate, $\tilde{\mu }_p$ is the rate of phagocytosis per foam cell per unit of available modLDL, and $\tilde{\mu }_c$ is the rate of efferocytosis per foam cell per unit of available dead material. Since $\tilde{s}_f + \tilde{s}_l + \tilde{s}_c = 0$, the model is locally mass conservative.

We assume that foam cells undergo undirected random motion (Owen and Sherratt 1997) and directed chemotactic motion toward modLDL. The latter is a simplification of the full process whereby modLDL stimulates the production of chemoattractants. Foam cells also undergo chemotactic motion toward dead material, in response to find-me signals expressed by the dying cells (Kojima et al 2017). ModLDL and dead material undergo diffusive motion. For simplicity, we assume constant diffusion coefficients for all phases, and linear chemotaxis with regards to modLDL and dead material for foam cells. This gives the flux terms

8 $$\begin{aligned} \tilde{J}_f&= - \tilde{D}_f \frac{\partial \tilde{f}}{\partial \tilde{x}} + \tilde{\chi }_l \tilde{f} \frac{\partial \tilde{l}}{\partial \tilde{x}} + \tilde{\chi }_c \tilde{f} \frac{\partial \tilde{c}}{\partial \tilde{x}} \,, \end{aligned}$$

9 $$\begin{aligned} \tilde{J}_l&= - \tilde{D}_l \frac{\partial \tilde{l}}{\partial \tilde{x}} \,, \end{aligned}$$

10 $$\begin{aligned} \tilde{J}_c&= - \tilde{D}_c \frac{\partial \tilde{c}}{\partial \tilde{x}} \,, \end{aligned}$$

where $\tilde{D}_f$ is the foam cell random motility coefficient, $\tilde{D}_l$ and $\tilde{D}_c$ are diffusion coefficients for modLDL and dead material, respectively, and $\tilde{\chi }_l$ and $\tilde{\chi }_c$ are chemotactic coefficients for foam cells in response to modLDL and dead material, respectively.

The endothelium permits a net influx of LDL carried by native LDL particles, which we assume is constant over plaque growth timescales of days or weeks. We also assume that typical timescales for chemical modification of LDL are much faster than plaque growth timescales (Cobbold et al 2002). Under this assumption, native LDL becomes inflammatory modLDL immediately after entering the intima, so we model native LDL deposition as an boundary modLDL influx. Monocyte recruitment requires adhesion molecules expressed by endothelial cells and chemokines produced in the intima. These are expressed in response to the presence of modified LDL. For simplicity, we assume monocyte recruitment is proportional to the amount of modLDL at the endothelium. We also assume that monocyte differentiation timescales are much faster than those for plaque growth (Blanchard et al 1991), and so we model monocyte recruitment as a macrophage boundary influx. Cells do not die until they are already inside the intima, so the flux of dead material at the endothelial boundary is zero. This gives the following flux boundary conditions at $\tilde{x} = 0$:

11 $$\begin{aligned} ( \tilde{J}_f + \tilde{v} \tilde{f} )&= \tilde{\sigma }_f \tilde{l} \,, \end{aligned}$$

12 $$\begin{aligned} ( \tilde{J}_l + \tilde{v} \tilde{l} )&= \tilde{\sigma }_l \,, \end{aligned}$$

13 $$\begin{aligned} ( \tilde{J}_c + \tilde{v} \tilde{c} )&= 0 \,, \end{aligned}$$

where $\tilde{\sigma }_l$ is the rate of LDL deposition and $\tilde{\sigma }_f$ is the rate of monocyte recruitment per unit of modLDL at the endothelium.

At the medial boundary, we assume zero flux boundary conditions for the passive modLDL and dead material phases. We assume foam cells will emigrate into the lymphatic vasculature at a rate proportional to the quantity of foam cells present. The corresponding flux boundary conditions at $\tilde{x} = \tilde{R}$ are given by

14 $$\begin{aligned} ( \tilde{J}_f + \tilde{v} \tilde{f} ) - \tilde{R}'(\tilde{t}) \tilde{f}&= \tilde{\sigma }_e \tilde{f} \,, \end{aligned}$$

15 $$\begin{aligned} ( \tilde{J}_l + \tilde{v} \tilde{l} ) - \tilde{R}'(\tilde{t}) \tilde{l}&= 0 \,, \end{aligned}$$

16 $$\begin{aligned} ( \tilde{J}_c + \tilde{v} \tilde{c} ) - \tilde{R}'(\tilde{t}) \tilde{c}&= 0 \,, \end{aligned}$$

where $\sigma _e$ is the average foam cell egress velocity.

For the initial conditions, we assume the intima is populated with a small number of live resident macrophages (Ley et al 2011), so

17 $$\begin{aligned} \tilde{f}(\tilde{x},0)&= N_0 \,, \end{aligned}$$

18 $$\begin{aligned} \tilde{l}(\tilde{x},0)&= 0 \,, \end{aligned}$$

19 $$\begin{aligned} \tilde{c}(\tilde{x},0)&= 0 \,, \end{aligned}$$

20 $$\begin{aligned} \tilde{R}(0)&= x_S \,, \end{aligned}$$

where $x_S$ is the diameter of a typical mouse macrophage.

In order to close the model, the mixture velocity $\tilde{v}(\tilde{x},\tilde{t})$ and the velocity of the medial boundary $\tilde{R}'(\tilde{t})$ need to be defined, in addition to initial conditions. For models with more than one spatial dimension that are not axisymmetric, additional assumptions are required in order to determine $\tilde{v}(\tilde{x},\tilde{t})$ and the boundary normal velocity. One possible choice is to relate the velocity to the pressure using Darcy’s law ($\tilde{v} = - k \nabla \tilde{P}$) (Friedman 2015; Lemon et al 2006). This requires additional constitutive assumptions and boundary conditions for the pressure *P*. Our model avoids these issues by only considering motion along the radial direction, and (1)–(16) are sufficient to close the system.

Summing over the phase continuity equations (1)–(3) and applying the no-voids condition (4) gives

21 $$\begin{aligned} 0 = - \frac{\partial }{\partial \tilde{x}} \left( { - \tilde{D}_f \frac{\partial \tilde{f}}{\partial \tilde{x}} + \tilde{\chi }_l \tilde{f} \frac{\partial \tilde{l}}{\partial \tilde{x}} + \tilde{\chi }_c \tilde{f} \frac{\partial \tilde{c}}{\partial \tilde{x}} - \tilde{D}_l \frac{\partial \tilde{l}}{\partial \tilde{x}} - \tilde{D}_c \frac{\partial \tilde{c}}{\partial \tilde{x}} + N_0 \tilde{v} }\right) \,. \end{aligned}$$

Integrating (21) from $\tilde{x} = 0$ to $\tilde{x}$ and substituting the $\tilde{x} = 0$ boundary conditions (11)–(13) gives the following expression for the mixture velocity:

22 $$\begin{aligned} \tilde{v} = \frac{1}{N_0} \left[ { \tilde{\sigma }_l + \tilde{\sigma }_f l \Big \vert _{\tilde{x}=0} + \tilde{D}_f \frac{\partial \tilde{f}}{\partial \tilde{x}} - \tilde{\chi }_l \tilde{f} \frac{\partial \tilde{l}}{\partial \tilde{x}} - \tilde{\chi }_c \tilde{f} \frac{\partial \tilde{c}}{\partial \tilde{x}} + \tilde{D}_l \frac{\partial \tilde{l}}{\partial \tilde{x}} + \tilde{D}_c \frac{\partial \tilde{c}}{\partial \tilde{x}} }\right] \,. \end{aligned}$$

The boundary velocity can be similarly obtained by integrating (21) from $\tilde{x} = 0$ to $\tilde{R}$ and substituting the $\tilde{x} = 0$ and $\tilde{R}$ boundary conditions (11)–(16), which yields

23 $$\begin{aligned} \frac{\textrm{d}\tilde{R}'}{\textrm{d}\tilde{t}} = \frac{1}{N_0} \left[ { \tilde{\sigma }_l + \tilde{\sigma }_f l \Big \vert _{\tilde{x}=0} - \tilde{\sigma }_e f \Big \vert _{\tilde{x}=\tilde{R}} }\right] \,. \end{aligned}$$

The full dimensional model consists of (1)–(20) and (23).

### Parameter Estimates and Model Nondimensionalisation

We nondimensionalise the model by applying the rescalings

24 $$\begin{aligned} f = \frac{\tilde{f}}{N_0} \,,\quad l = \frac{\tilde{l}}{N_0} \,,\quad c = \frac{\tilde{l}}{N_0} \,,\quad t = \frac{\tilde{t}}{t_S} \,,\quad x = \frac{\tilde{x}}{x_S} \,, \end{aligned}$$

where $t_S$ and $x_S$ are some reference timescale and length scale, and $N_0$ is the total phase density.

We choose $t_S$ to be one week as experimental studies on plaques in mouse models typically take place over periods of weeks to months (Williams et al 2018; Potteaux et al 2011). Lengths are rescaled by the diameter of a typical mouse macrophage. As observed macrophage sizes vary from under $14\,\mu \hbox {m}$ for murine bone marrow-derived macrophages (Cannon and Swanson 1992) to $20\,\mu \hbox {m}$ for murine peritoneal macrophages (Nguyen et al 2012), we choose a characteristic size of $16\,\mu \hbox {m}$. Estimating certain parameters requires the conversion of three-dimensional volume densities to one-dimensional linear densities. For the purposes of parameter estimation, we consider a cylindrical section through the intima whose circular cross section is transverse to the positive *x* vector and has diameter $x_S$, so that the maximum linear phase density $N_0$ is one cell per unit of $x_S$.

Foam cell motility parameters $\tilde{D}_f$ and $\tilde{\chi }_l$ are estimated using models fitted to data from chemotaxis assay experiments (Owen and Sherratt 1997; Sozzani et al 1991). The phagocytosis parameter $\tilde{\mu }_p$ is based on experimental observations where macrophages are observed to phagocytose a significant volume of modLDL after a timescale of approximately 60 min (Zwaka et al 2001). The boundary monocyte recruitment parameter $\tilde{\sigma }_f$ is based on an experimental study by Jeng et al (1993) where monocytes pretreated with modLDL are exposed to shear flow and are observed to bind to an endothelial cell monolayer with an area of $0.1452\,\hbox {mm}^{2}$ at a rate of 140–200 cells over 30 min. The death rate $\tilde{\mu }_a$ is based on experimental observations of mouse macrophages incubated in acetylated LDL, where $12\%$ of the initial macrophage population is observed to undergo apoptosis after ${9}\,{\hbox {h}}$. Fitting this to a simple exponential decay curve gives an apoptosis parameter of about ${0.25}\,{\hbox {h}^{-1}}$ before nondimensionalising.

The remaining parameters are based on order of magnitude estimates. The modLDL diffusion coefficient $\tilde{D}_l$ is estimated to be larger than $\tilde{D}_f$ due to the small size of LDL particles compared to macrophages. Experimental studies of LDL phagocytosis (Zwaka et al 2001; Suits et al 1989) find that for heavily cholesterol-laden foam cells, cholesterol droplets comprise a significant proportion of the total foam cell volume, and so the range of LDL influx rates is chosen so that $\tilde{\sigma }_l$ is of a similar order of magnitude to the monocyte influx $\tilde{\sigma }_f \tilde{l}|_{\tilde{x}=0}$. The upper range of foam cell egress velocities $\tilde{\sigma }_e$ is chosen so that the medial egress flux $\tilde{\sigma }_e \tilde{f}|_{\tilde{x}=\tilde{R}}$ is of a similar order of magnitude to the endothelial LDL and monocyte fluxes $\tilde{\sigma }_l$ and $\tilde{\sigma }_f \tilde{l}|_{\tilde{x}=0}$. For efferocytosis, we choose a range of values for $\tilde{\mu }_e$ for which efferocytic uptake rates are of a similar order of magnitude to apoptosis rates. Dead material diffusion is assumed to be slower than live macrophage random motility due to the lack of active locomotion. We choose $\tilde{D}_c$ to be lower than $\tilde{D}_f$ within an order of magnitude. The macrophage chemotactic response to dead material $\tilde{\chi }_c$ is assumed to be lower than that for modLDL, since the dead material phase includes necrotic material which expresses few find-me signals.

Tables 1 and 2 summarise the model parameter values before and after rescaling.

**Table 1:** Dimensional parameter estimates (order of magnitude estimates used where quantitative biological data was unavailable)

Parameter + Description		Dimensional estimate	Sources
$t_S$	reference time scale	6$\cdot 10^{5}$ s	−
$x_S$	reference length scale	1.6$\cdot 10^{-5}$ m	(Cannon and Swanson 1992)
$N_0$	maximum phase density	${1}\,{\hbox {cell}}/x_S$	−
$\tilde{D}_f$	foam cell random motility coefficient	$4\cdot 10^{-15}-10^{-14}\hbox {m}^{2}\hbox {s}^{-1}$	(Owen and Sherratt 1997)
$\tilde{D}_l$	modLDL diffusion coefficient	$> \tilde{D}_f$	order of mag est
$\tilde{D}_c$	dead material diffusion coefficient	$< \tilde{D}_f$	order of mag est
$\tilde{\chi }_l$	foam cell chemotactic coefficient (modLDL)	$\gg \tilde{D}_f/N_0$	order of mag est, (Owen and Sherratt 1997)
$\tilde{\chi }_c$	foam cell chemotactic coefficient (dead material)	$\sim \tilde{\chi }_l$	order of mag est
$\tilde{\mu }_a$	foam cell death rate	$\sim {0.25}\,\hbox {h}^{-1}$	(Yao and Tabas 2000)
$\tilde{\mu }_e$	efferocytosis rate	$\sim \tilde{\mu }_a / N_0$	order of mag est, (Yao and Tabas 2000)
$\tilde{\mu }_p$	modLDL phagocytosis rate	$\sim ({30}\,{\hbox {min}})^{-1}/N_0$	(Zwaka et al 2001)
$\tilde{\sigma }_f$	monocyte recruitment rate per unit modLDL	$\sim 5\cdot 10^{-5}\,\hbox {cell\,m}^{-2}s^{-1} \cdot x_S^2 /N_0$	(Jeng et al 1993)
$\tilde{\sigma }_l$	LDL deposition rate	$\sim \tilde{\sigma }_f N_0$	order of mag est
$\tilde{\sigma }_e$	foam cell egress velocity	$\sim \tilde{\sigma }_f,\, \tilde{\sigma }_l/N_0$	order of mag est

**Table 2:** Rescaled parameters for the nondimensionalised model

Parameter	Rescaling	Nondimensional estimate
$D_f$	$\tilde{D}_f/(x_S^2/t_S)$	20
$D_l$	$\tilde{D}_l/(x_S^2/t_S)$	200
$D_c$	$\tilde{D}_c/(x_S^2/t_S)$	10
$\chi _l$	$\tilde{\chi }_l/(x_S^2/N_0 t_S)$	1000
$\chi _c$	$\tilde{\chi }_c/(x_S^2/N_0 t_S)$	500
$\mu _a$	$\tilde{\mu }_a/(1/t_S)$	${10}{-}{100}$ (variable)
$\mu _e$	$\tilde{\mu }_e/(1/N_0 t_S)$	${10}-{200}$ (variable)
$\mu _p$	$\tilde{\mu }_p/(1/N_0 t_S)$	300
$\sigma _f$	$\tilde{\sigma }_f/(x_S/t_S)$	100
$\sigma _l$	$\tilde{\sigma }_l/(N_0 x_S/t_S)$	10
$\sigma _e$	$\tilde{\sigma }_e/(x_S/t_S)$	${10}-{100}$ (variable)

The final nondimensional model consists of the constitutive equations

25 $$\begin{aligned} \frac{\partial f}{\partial t}&= -\frac{\partial }{\partial x} (J_f + v f) + s_f \,, \end{aligned}$$

26 $$\begin{aligned} \frac{\partial l}{\partial t}&= -\frac{\partial }{\partial x} (J_l + v l) + s_l \,, \end{aligned}$$

27 $$\begin{aligned} \frac{\partial c}{\partial t}&= -\frac{\partial }{\partial x} (J_c + v c) + s_c \,, \end{aligned}$$

with no-voids condition

28 $$\begin{aligned} f + l + c = 1 \,, \end{aligned}$$

source terms

29 $$\begin{aligned} s_f&= + \mu _p f l + \mu _e f c - \mu _a f \,, \end{aligned}$$

30 $$\begin{aligned} s_l&= - \mu _p f l \,, \end{aligned}$$

31 $$\begin{aligned} s_c&= - \mu _e f c + \mu _a f \,, \end{aligned}$$

flux terms

32 $$\begin{aligned} J_f&= - D_f \frac{\partial f}{\partial x} + \chi _l f \frac{\partial l}{\partial x} + \chi _c f \frac{\partial c}{\partial x} \,, \end{aligned}$$

33 $$\begin{aligned} J_l&= - D_l \frac{\partial l}{\partial x} \,, \end{aligned}$$

34 $$\begin{aligned} J_c&= - D_c \frac{\partial c}{\partial x} \,, \end{aligned}$$

boundary conditions at $x = 0$

35 $$\begin{aligned} ( J_f + v f )&= \sigma _f l \,, \end{aligned}$$

36 $$\begin{aligned} ( J_l + v l )&= \sigma _l \,, \end{aligned}$$

37 $$\begin{aligned} ( J_c + v c )&= 0 \,, \end{aligned}$$

boundary conditions at $x = R$

38 $$\begin{aligned} ( J_f + v f ) - R' f&= \sigma _e f \,, \end{aligned}$$

39 $$\begin{aligned} ( J_l + v l ) - R' l&= 0 \,, \end{aligned}$$

40 $$\begin{aligned} ( J_c + v c ) - R' c&= 0 \,, \end{aligned}$$

and initial conditions

41 $$\begin{aligned} f(x,0)&= 1 \,, \end{aligned}$$

42 $$\begin{aligned} l(x,0)&= 0 \,, \end{aligned}$$

43 $$\begin{aligned} c(x,0)&= 0 \,, \end{aligned}$$

44 $$\begin{aligned} R(0)&= 1 \,, \end{aligned}$$

where the mixture velocity is

45 $$\begin{aligned} v = \sigma _f l \Big \vert _{x=0} + \sigma _l + D_f \frac{\partial f}{\partial x} - \chi _l f \frac{\partial l}{\partial x} - \chi _c f \frac{\partial c}{\partial x} + D_l \frac{\partial l}{\partial x} + D_c \frac{\partial c}{\partial x} \,. \end{aligned}$$

and the domain grows at rate

46 $$\begin{aligned} \frac{{\textrm{d}}R}{{\textrm{d}}t} = \sigma _f l \Big \vert _{x=0} + \sigma _l - \sigma _e f \Big \vert _{x=R}\,. \end{aligned}$$

### Numerical Solution

The system was solved numerically using the method of lines. The nondimensional system was first mapped onto a fixed spatial domain [0, 1] using the transformation $(x,t) \rightarrow (y,\tau ) = (x/R(t),t)$, thereby transforming it into a system of parabolic PDEs coupled to an ODE for the domain length $R(\tau )$. The transformed system was then reduced to a set of time-dependent ODEs by discretising spatial derivatives using a central differencing approximation. The resulting ODEs were solved using a backward differentiation formula method in Python using SciPy’s integration libraries.

## Plaque Structure, Cell Death, and Efferocytosis

In this section, we neglect emigration, setting $\sigma _e = 0$, and focus on how the relative rates of cell death and efferocytosis influence the composition of the plaque.

### Base Case Simulation

**Fig. 2:**
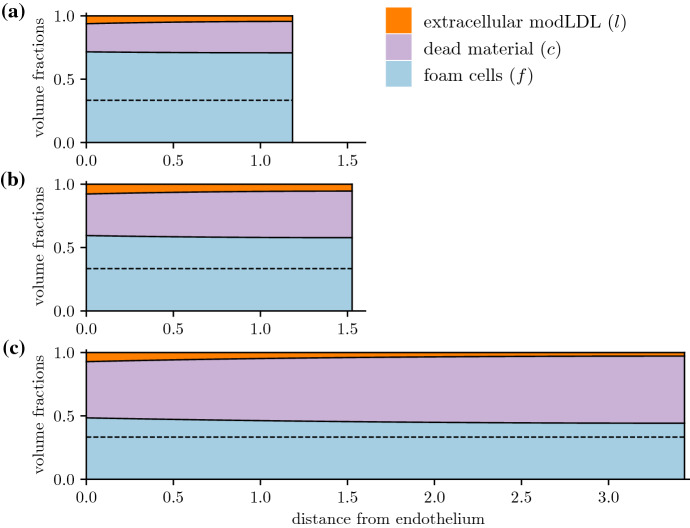
Early time evolution of a plaque’s structure with $\mu _a = 40$, $\mu _e = 60$, $\sigma _e = 0$, plotted at times **a** $t = 0.01$, **b** $t = 0.025$, **c** $t = 0.1$. The dotted line denotes the $f^* = 1 - \tfrac{\mu _a}{\mu _e} = 0.333$ steady-state foam cell density predicted by the ODE model. Figure 4a plots the total quantities of each phase as well as the plaque size as they vary with time

**Fig. 3:**
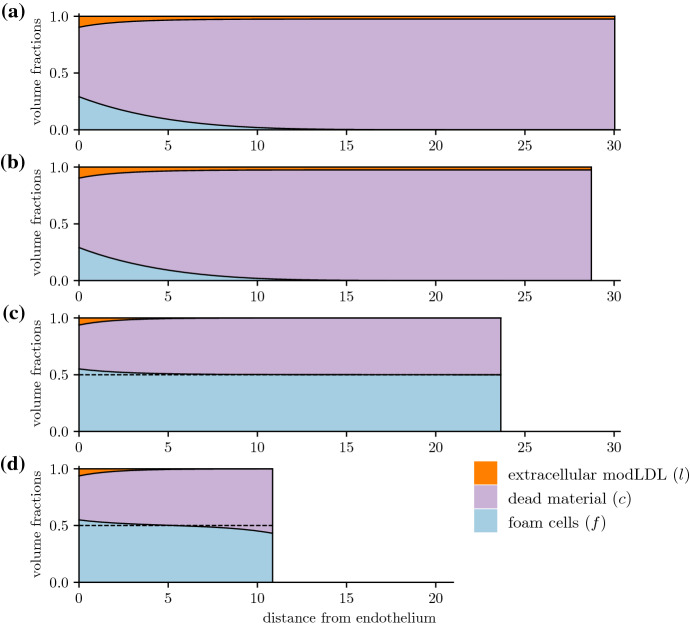
Comparisons of plaque structure at $t = 1$ for $\mu _a = 40$ and **a** $\mu _e = 20$, $\sigma _e = 0$, **b** $\mu _e = 20$, $\sigma _e = 30$, **c** $\mu _e = 80$, $\sigma _e = 0$, **d** $\mu _e = 80$, $\sigma _e = 30$. The dotted lines in **c** and **d** denote the $f^* = 1 - \tfrac{\mu _a}{\mu _e} = 0.5$ steady-state foam cell density predicted by the ODE model (for **a** and **b**, an $f^* = 0$ steady state is predicted)

Figure 2 illustrates the evolution of a base case with relatively high efferocytic uptake compared to cell death. Following the initial injury where LDL starts being deposited in the intima, the plaque quickly reaches a state where it is populated almost entirely by foam cells and dead material, although the balance between the two may vary (Fig. 2). A small amount of modLDL that has yet to be ingested remains near the endothelium, though some uningested modLDL may remain throughout the intima for very early times (Fig. 2), or for cases where all foam cells in the deep plaque have died (Fig. 3a). ModLDL and live foam cell densities are higher near the endothelium than in the deep plaque due to the constant influx of new monocytes and LDL particles, though some dead material is still present due to diffusion. Away from the endothelium, phase densities are largely uniform, with some non-uniformity observed near the medial boundary when $\sigma _e > 0$ (Fig. 3d).

**Fig. 4:**
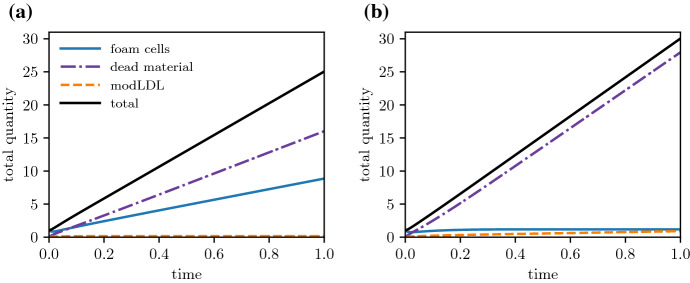
Time evolution of the total phase constituents $\int _0^R u \textrm{d}x$ (for phases $u = f,l,c$) immediately following plaque initialisation. Plots are for plaques with $\mu _a = 40$, $\sigma _e = 0$, and **a** $\mu _e = 60$, **b** $\mu _e = 20$

The intima itself quickly approaches a state where it grows at a constant rate (Fig. 4). The rates at which foam cells and dead material accumulate also settle to a constant rate, while uningested modLDL may either reach a steady value or accumulate at a constant rate, depending on whether the deep plaque still has live foam cells remaining to ingest new lipid (Fig. 4a vs. b).

We remark that increasing the LDL deposition rate $\sigma _l$ or the monocyte recruitment parameter $\sigma _f$ will increase the intimal growth rate. This is due to the increased influx of new material in the form of modLDL or recruited monocytes, respectively. As we see later however, the qualitative spatial structure depends primarily on the cell death and efferocytosis rates, which remain our focus in this section.

**Fig. 5:**
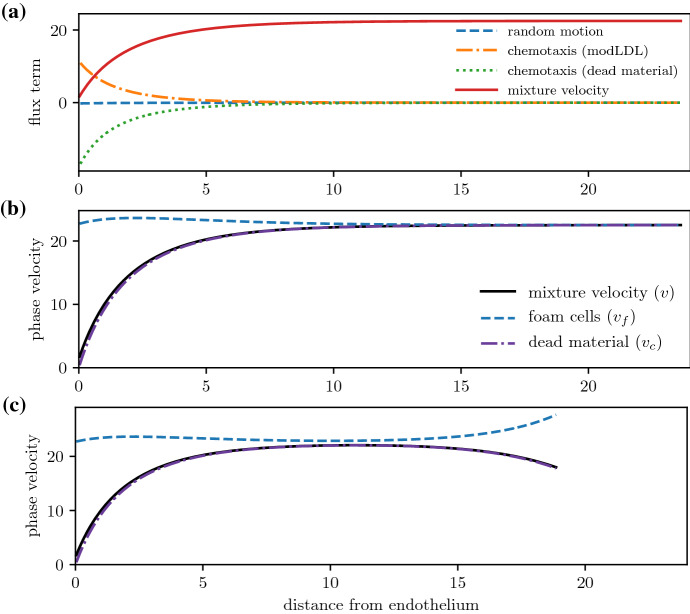
**a** Comparison of the individual flux terms in the constitutive equation (25) and (29))) for the foam cell phase: random motion $-D_f \frac{\partial f}{\partial x}$, chemotaxis toward modLDL $\chi _l f \frac{\partial l}{\partial x}$ and dead material $\chi _c f \frac{\partial c}{\partial x}$, and bulk advection *vf*. **b**, **c** Comparison of the foam cell and dead material phase velocities with the mixture velocity. Terms are plotted as a function of *x* at a fixed time $t = 1$ for a scenario with $\sigma _a = 40$ and **b** $\sigma _e = 0$, **c** $\sigma _e = 80$

Figure 5a compares the relative sizes of the diffusive, chemotactic, and advective foam cell flux terms. Diffusion and chemotaxis are dominated by advective transport, which is the sole driver of foam cell movement in the deep plaque. Chemotaxis is important near the endothelium where there are larger amounts of uningested modLDL, but becomes irrelevant in the deeper plaque once the available modLDL has been internalised. From Fig. 5b, the total phase velocities for both the foam cell and dead cell phases converge to the mixture velocity in the deep plaque, with little interphase motion. This suggests that mass transport in the deep plaque occurs primarily via bulk advection. Far from the boundary, the system may therefore be approximated as a advection-reaction equation by dropping the much smaller diffusive and chemotactic terms. We retain these terms for all numerical simulations in this paper due to the discrepancy between the phase velocities at the endothelial boundary, and to avoid numerical instabilities that may arise by dropping the diffusive term.

We note also that when $\sigma _e > 0$ (Fig. 5c), emigration of foam cells into the lymphatics causes some deviation between the mixture velocity and the foam cell phase velocity at $x=R$. Nevertheless, the $\sigma _e = 0$ case provides a good baseline for discussing phase densities, since the close agreement between phase velocities away from $x=0$ makes the system amenable to analysis using the method of characteristics.

### ODE Approximation for Deep Plaque Composition

The constitutive equations for each phase *u* can be written in the form

47 $$\begin{aligned} \frac{\partial u}{\partial t} + \frac{\partial }{\partial x} (v_u u) = s_u \,, \end{aligned}$$

where $v_u = (J_u + v u)/u$ is the total velocity of phase *u* and $s_u$ is its source term. In Fig. 5, we observed that away from the endothelium, the bulk advection term *vu* dominates over random and chemotactic motion modelled by $J_u$ for all three phases, and so $v_u \approx v$ in the deep plaque. For each phase *u*, we define $U_i(t)$ to be the phase density along a characteristic curve $x_c(t)$ advected with speed $v_u$, i.e.

48 $$\begin{aligned} U(t) = u( x_c(t), t ) \,,\quad \frac{{\textrm{d}}x_c}{{\textrm{d}}t} = v_u \,. \end{aligned}$$

In the deep plaque, $v_u \approx v$ for all phases, and we can make the approximation

49 $$\begin{aligned} \frac{{\textrm{d}}U}{{\textrm{d}}t} \approx s_u \,. \end{aligned}$$

We note also that in the deep plaque, the modLDL density *l*(*x*, *t*) is negligible, so we make an additional approximation $l = 0$. Substituting the model source terms (29) and (30) in (49) yields the ODE system

50 $$\begin{aligned} \frac{{\textrm{d}}F}{{\textrm{d}}t}&= - \mu _a F + \mu _e F C \,, \end{aligned}$$

51 $$\begin{aligned} \frac{{\textrm{d}}C}{{\textrm{d}}t}&= + \mu _a F - \mu _e F C \,, \end{aligned}$$

where $F(t) = f(x_c(t),t)$ and $C(t) = c(x_c(t),t)$ denote the phase densities along characteristic curves. Due to the no-voids condition (28), we also have $F + C = 1$. Using this condition, the coupled equations reduce to a single ODE

52 $$\begin{aligned} \frac{{\textrm{d}}F}{{\textrm{d}}t} = \mu _e F \left( {{1-\tfrac{\mu _a}{\mu _e}} - F }\,\right) . \end{aligned}$$

This equation has two steady states at $F^* = 0$ and $F^* = 1-\tfrac{\mu _a}{\mu _e}$, with a transcritical bifurcation occurring when $\mu _a = \mu _e$. Linearising about the steady states gives eigenvalues of $\lambda = \mu _e - \mu _a$ at $F^* = 0$ and $\lambda = \mu _a - \mu _e$ at $F^* = 1 - \frac{\mu _a}{\mu _e}$.

When $\mu _a < \mu _e$ and efferocytosis rates are high relative to cell death, $F^* = 1-\tfrac{\mu _a}{\mu _e}$ is the sole stable steady state, and the system tends toward a state where live foam cells coexist with dead material. When $\mu _a < \mu _e$ however, $F^* = 0$ is the sole stable state. Here, efferocytic recycling is insufficient to counterbalance cell death, and the plaque settles to a state where all foam cells die off. From the eigenvalues, for a fixed ratio $\frac{\mu _a}{\mu _e}$, higher overall rates of cell death and efferocytosis will cause the system to settle to the relevant steady state faster.

### Plaque Composition and the Death/Efferocytosis Balance

**Fig. 6:**
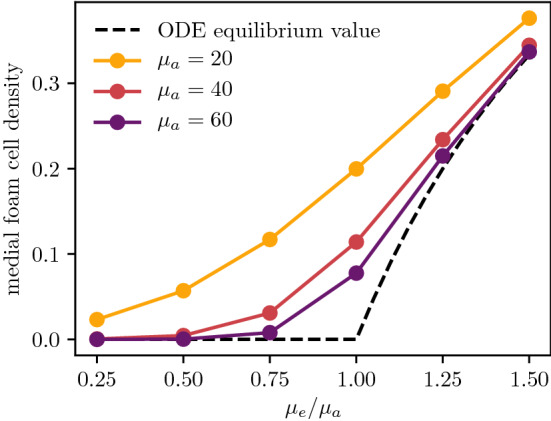
Comparison of the deep plaque foam cell density $f|_{x=R}$ as a function of the efferocytosis/death ratio $\tfrac{\mu _e}{\mu _a}$, plotted at $t = 0.5$ for various values of $\mu _a$. The equilibrium density predicted by the ODE model is also given for comparison

The deep plaque composition closely matches the steady-state densities predicted by the ODE model. In Fig. 3, lower rates of efferocytosis are associated with higher amounts of dead material and fewer living foam cells. For plaques with efficient efferocytosis where $\mu _e > \mu _a$ (Fig. 3c and d), the foam cell density settles to the $f^* = 1 - \frac{\mu _a}{\mu _e}$ density predicted by the ODE model. On the other side of the $\tfrac{\mu _e}{\mu _a} = 1$ bifurcation point, plaques with $\mu _e < \mu _a$ (Fig. 3a and b) reach a state where the deep plaque supports no live foam cells. For especially low values of $\mu _e$ (Fig. 3a), foam cells will die before ingesting all available extracellular modLDL, leaving some remnant modLDL which gets carried advectively into the deep plaque. From Fig. 6, higher rates of death and efferocytosis for a given value of $\frac{\mu _a}{\mu _e}$ lead to better agreement between the ODE steady states and the deep plaque densities at $x = R$. This is consistent with linear analysis of the ODE system, which predicts faster convergence to the steady state for higher values of $\mu _a$ and $\mu _e$.

**Fig. 7:**
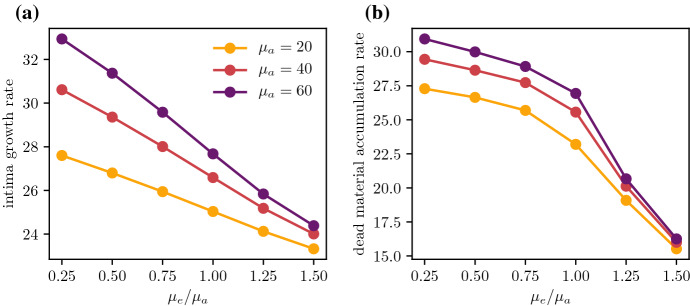
Comparison of **a** the intima growth rate $\frac{\textrm{d}R}{\textrm{d}t}$ and **b** the rate of change of total dead material $\frac{\textrm{d}}{\textrm{d}t} \int _0^R c \,\textrm{d}x$ as a function of the efferocytosis/death ratio $\tfrac{\mu _e}{\mu _a}$, plotted at $t = 1$ for various values of $\mu _a$

Cell death and efferocytosis also influence the growth rate of a plaque, even with fixed values of the monocyte and LDL influx parameters $\sigma _f$ and $\sigma _l$. From Fig. 7a, increasing $\mu _e$ with fixed $\mu _a$ will slow the rate of growth. This is because increasing the efficiency of efferocytosis will increase the numbers of foam cells actively consuming modLDL, thus reducing endothelial modLDL levels and dulling the inflammatory response. This slowing in growth can also be observed when comparing plaque sizes in Fig. 3. Reducing $\mu _e$ and $\mu _a$ with constant $\frac{\mu _e}{\mu _a}$ has the same effect of slowing plaque growth by reducing endothelial modLDL levels, since reducing the rate at which cells start to die will increase the quantity of available foam cells near the endothelium.

The rate at which a plaque accumulates new dead material is a useful marker of plaque health alongside its total growth rate. Both are low in a healthy plaque. As discussed previously, reducing $\mu _e$ and $\mu _a$ with constant $\frac{\mu _e}{\mu _a}$ reduces the rate at which new cellular material enters the plaque. From Fig. 7b, this results in a slight decrease in the total rate of increase of dead material, although the proportion of dead material in the bulk of the plaque remains the same. Increasing the efficiency of efferocytosis via $\mu _e$ with constant $\mu _a$ has a more significant effect on the rate of accumulation, as it reduces both the monocyte influx and the local dead cell proportion in the interior of the plaque by promoting more active uptake of dead material.

## Emigration and Plaque Growth

Foam cell emigration can slow plaque growth and potentially allow plaque size to stabilise by allowing material to leave the plaque. The ability of emigration to impact plaque growth however depends on the availability of live foam cells.

**Fig. 8:**
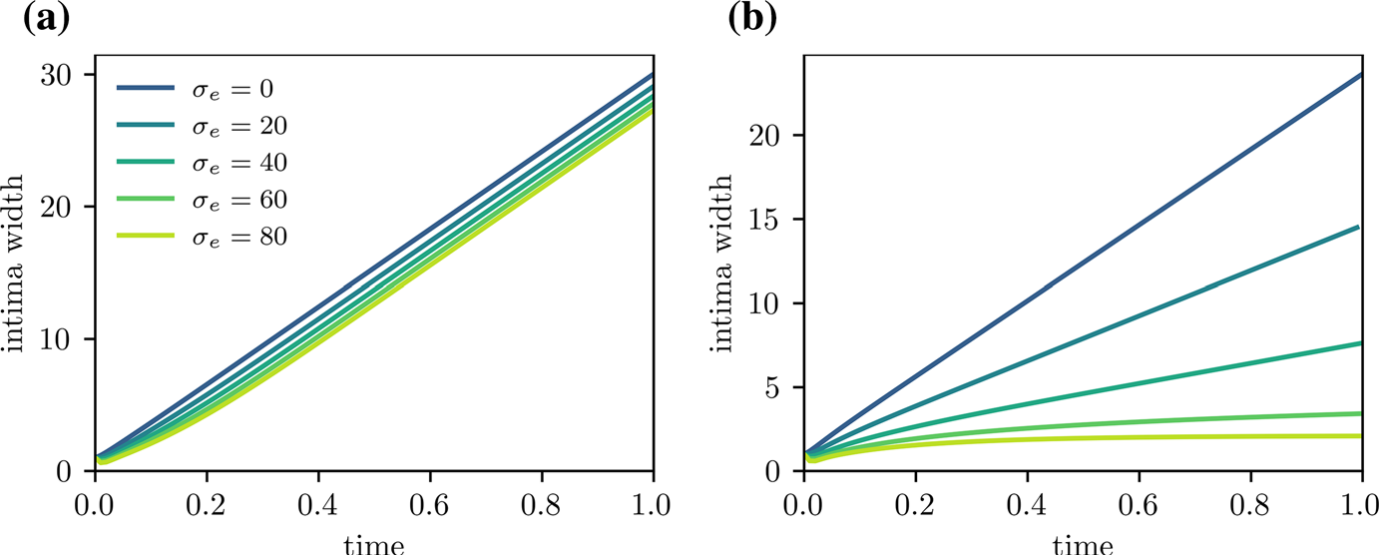
Plaque growth for varying egress velocities $\sigma _e$, for low and high $\tfrac{\mu _e}{\mu _a}$ scenarios. Plots track the intima width *R*(*t*), where $\mu _a = 40$, and **a** $\mu _e = 20$, **b** $\mu _e = 80$

In Figs. 3a and b and 8a, $\mu _e < \mu _a$, and efferocytosis is insufficient to prevent the death of foam cells in the deep plaque. Increasing the emigration velocity here has little effect on plaque size as there are few foam cells close to the medial edge of the plaque. In Fig. 3c and d on the other hand, $\mu _e > \mu _a$, and there is a higher density of foam cells in the deep plaque. Increasing the emigration velocity here slows the rate of plaque growth by enabling material to leave the plaque, which also results a reduction in plaque size. For sufficiently high $\sigma _e$, the plaque will stabilise entirely and settle to a steady state with no growth (Fig. 8b). While emigration does cause a slight reduction in foam cells numbers near the media, their numbers remain sufficient to allow emigration. We remark briefly that emigration causes phase densities near $x = R$ to deviate from those predicted by the ODE model, which we discuss further in Sect. 6.

**Fig. 9:**
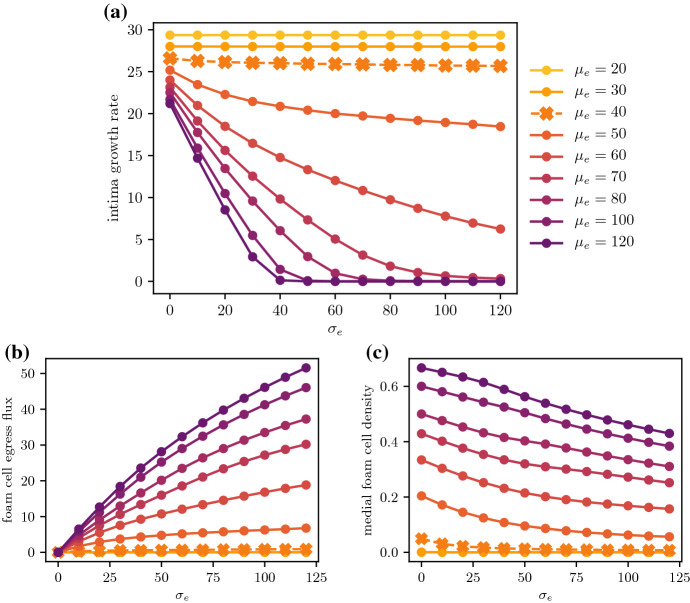
Plots showing the effect of the emigration velocity $\sigma _e$ on plaque development, compared across various efferocytosis rates $\mu _e$ with $\mu _a = 40$ fixed. Quantities are measured at $t = 1$. Plots compare **a** the total intima growth rate $\frac{\textrm{d}R}{\textrm{d}t}$, **b** the total flux of foam cells out of the medial boundary $j_f|_{x=R}$ via emigration, and **c** the foam cell density at the medial boundary $f|_{x=R}$

The importance of healthy efferocytosis in enabling foam cell emigration is more apparent when considering a range of values of $\mu _e$. From Fig. 9b, increasing the emigration velocity $\sigma _e$ will increase the total foam cell emigration flux for most values of $\mu _e$. This flux is diminished for lower values of $\mu _e$, and for $\mu _e \le \mu _a$, foam cell emigration remains negligible even for very high values of $\sigma _e$. Figure 9c suggests that for cases where when $\mu _e \le \mu _a$, the lack of emigration observed is due to the absence of live foam cells in the deep plaque. Conversely, cases with $\mu _e > \mu _a$ will still have some foam cells near the media, thus enabling emigration. Even for these cases however, higher emigration velocities result in lower medial foam cell densities due to the faster removal of foam cells. In particular, for cases where $\mu _e > \mu _a$ with $\mu _e$ close to $\mu _a$, the low live foam cell density gives diminishing returns on the total emigration flux when the emigration velocity is increased.

These results suggest that cell death will interfere with or prevent plaque stabilisation via foam cell emigration, depending on how efficiently efferocytosis acts. From Fig. 9a, for $\mu _e \le \mu _a$, increasing $\sigma _e$ has no effect on the plaque growth rate as there are no foam cells to emigrate from the plaque. For values of $\mu _e > \mu _a$ close to $\mu _a$, emigration is able to slow plaque growth, but full plaque stabilisation is not observed even for very high values of $\sigma _e$. For higher values of $\mu _e$, stabilisation is observed for high enough $\sigma _e$, with higher $\mu _e$ scenarios achieving this more readily. We briefly note that the endothelial LDL and monocyte influx parameters $\sigma _l$ and $\sigma _f$ also affect plaque growth in the presence of emigration. This is because higher $\sigma _l$ and $\sigma _f$ necessitate higher emigration rates $\sigma _e$ to balance the higher rates of material influx at the endothelium.

## Macrophage Circulation and Retention

In this section, we investigate the movement and retention of macrophage foam cells in the intima. We do so by extending the model to include an additional bead species that moves with foam cells (or dead cells), based on experimental work that labels and tracks macrophages using fluorescent latex microspheres (Williams et al 2018).

### Extending the Model: Bead Tagging

For the bead labelling model, we add two new species $q_f$ and $q_c$, representing beads carried by live foam cells and dead material, respectively. We assume that the volume occupied by the beads is negligible and that their densities can be modelled by the continuity equations

53 $$\begin{aligned} \frac{\partial q_f}{\partial t}&= -\frac{\partial }{\partial x} (J_{q_f} + v q_f) + s_{q_f} \,, \end{aligned}$$

54 $$\begin{aligned} \frac{\partial q_c}{\partial t}&= -\frac{\partial }{\partial x} (J_{q_c} + v q_c) + s_{q_c} \,. \end{aligned}$$

We suppose that beads are transported with the same velocity as the phase in which they are being carried. Using the flux-velocity relation $v_u u = j_u \,(= J_u + v u)$ gives the bead flux terms

55 $$\begin{aligned} J_{q_f}&= \frac{q_f}{m} J_f \,,\quad J_{q_c} = \frac{q_c}{c} J_c \,. \end{aligned}$$

Beads carried by macrophages are released into the dead material phase at the rate $\mu _a$ with which foam cells die. Similarly, beads within dead material are taken up by foam cells via efferocytosis at the same rate $\mu _e m$ as dead material is taken up. This gives the bead source terms

56 $$\begin{aligned} s_{q_f}&= -\, \mu _a q_f + \mu _e m q_c \,, \end{aligned}$$

57 $$\begin{aligned} s_{q_c}&= +\, \mu _a q_f - \mu _e m q_c \,. \end{aligned}$$

Note that bead numbers are locally conserved since $s_{q_f} + s_{q_c} = 0$.

At the endothelial boundary, we assume that macrophage-carried beads are introduced over a brief time period centred at $t_{\text {tag}}$. We model the bead influx with a Gaussian function, so that bead counts rise and fall over approximately 6 hours ($t = 0.035$). We represent this via the following bead endothelial boundary conditions:

58 $$\begin{aligned} (J_{q_f} + v q_f) \Big \vert _{x=0}&= \exp \left( { -\tfrac{1}{2} (\tfrac{t-t_{\text {tag}}}{0.035})^2 }\right) \,, \end{aligned}$$

59 $$\begin{aligned} (J_{q_c} + v q_c) \Big \vert _{x=0}&= 0 \,. \end{aligned}$$

The corresponding medial boundary conditions are

60 $$\begin{aligned} (J_{q_f} + v q_f) \Big \vert _{x=R} - R' q_f&= \sigma _e q_f \Big \vert _{x=R} \,, \end{aligned}$$

61 $$\begin{aligned} (J_{q_c} + v q_c) \Big \vert _{x=R} - R' q_c&= 0 \,. \end{aligned}$$

These equations can be derived from (55) and (16).

### Results

**Fig. 10:**
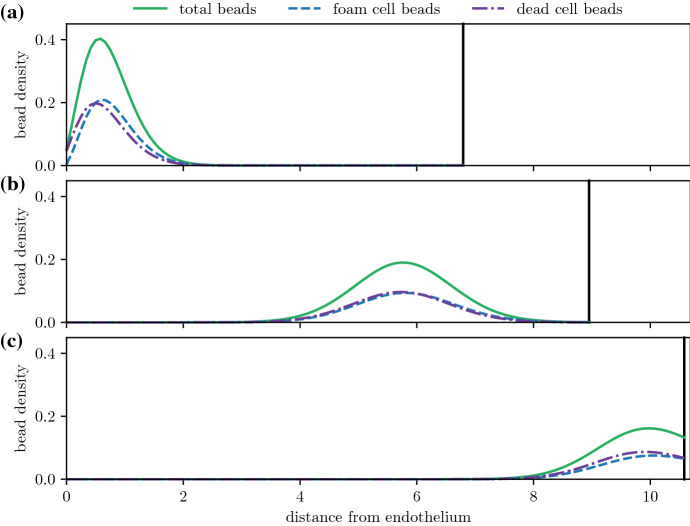
Time evolution of the bead spatial distribution for a plaque with high efferocytosis ($\mu _a =40$, $\mu _e = 80$, $\mu _e =20$), where the bead tag dosage is centred at time $t_{\text {tag}} = 1.0$. Distributions are plotted at times **a** $t = 1.1$, **b** $t = 1.5$, and **c** $t = 1.8$. The solid black line denotes the medial plaque boundary $x=R(t)$

**Fig. 11:**
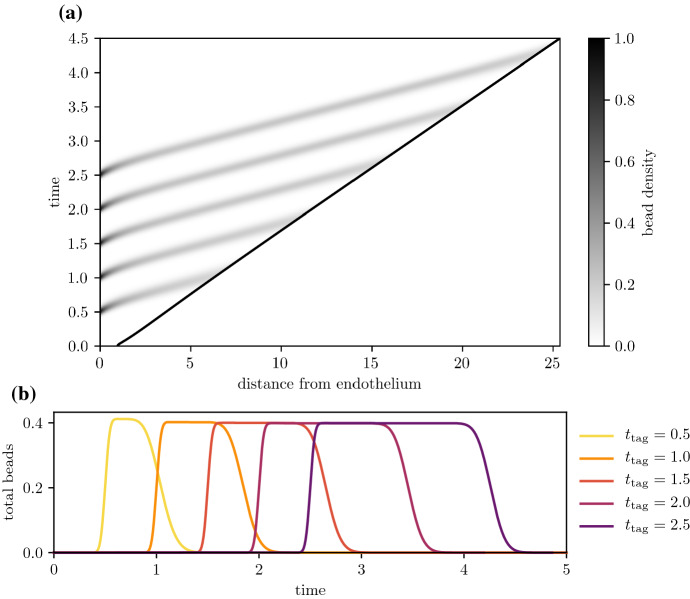
Bead circulation for different bead tagging times for a plaque with high efferocytosis ($\mu _a = 40$, $\mu _e = 80$, $\sigma _e = 20$). Plot **a** shows the time evolution of the total bead distribution $q_f(x,t) + q_c(x,t)$, where beads are administered multiple times at $t_{\text {tag}} = 0.5,\, 1.0,\, 1.5,\, 2.0,$ and 2.5. Plot **b** compares the time evolution of the total amount of beads $Q(t) = \int _0^{R(t)} (q_f+q_c) \textrm{d}x$ for each separate bead dosage

For plaques with sufficiently high foam cell emigration, foam cells will move closer to the medial boundary and eventually reach it, before emigrating into the arterial lymphatics. Figures 10 and 11a show the time evolution of the bead distribution in a plaque with nonzero emigration ($\sigma _e = 20$) and efferocytosis rates that allow live foam cells to persist deep into the plaque ($\mu _a = 40$, $\mu _e = 80$, so $\frac{\mu _e}{\mu _a}=2$). After bead-carrying monocytes have been recruited, the bead population is pushed deeper into the plaque as the plaque grows. Despite the increasing plaque size, the distance from the bead population to the medial boundary decreases due to material exiting through the boundary.

The amount of time that foam cells spend in the intima depends on emigration rates and the size of the plaque. Figure 11 considers the same high efferocytosis plaque scenario as Fig. 10, but with bead tagging happening at different times. From Fig. 11a, as the plaque increases in size, tagged foam cells that are recruited later have to travel a greater distance before they reach the medial boundary. As a result, they spend more time in the plaque. Figure 11b shows the time evolution of the total amount of beads

62 $$\begin{aligned} Q(t) = \int _0^{R(t)} (q_f(x,t) + q_c(x,t)) \textrm{d}x \,. \end{aligned}$$

**Fig. 12:**
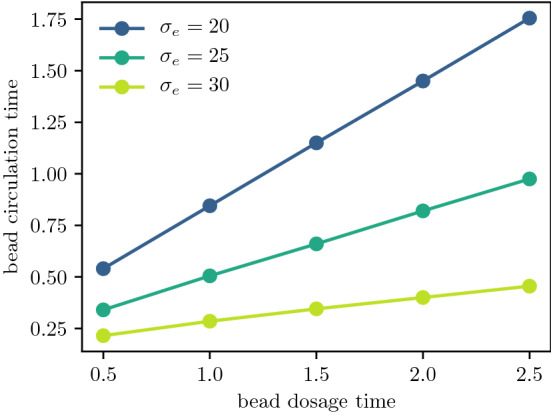
Total bead circulation time $t_{\text {circ}}$ for varying bead tagging times $t_{\text {tag}}$, plotted for different emigration velocities $\sigma _e$. Plaque parameters used are $\mu _a = 40$, $\mu _e = 80$

Bead totals peak soon after the tagging time $t_{\text {tag}}$, and fall to 0 when tagged foam cells emigrate from the intima. Bead-tagged foam cells that are recruited later spend more time in the intima. Figure 12 shows how the total circulation time of tagged monocytes depends on the time of recruitment and the average emigration velocity. The total circulation time $t_{\text {circ}}$ is estimated using the temporal full width at half maximum duration of the peak bead quantity, i.e.

63 $$\begin{aligned} t_{\text {circ}} = \max \left\{ t \,|\, Q(t) \ge 0.5 \, \max _t Q(t)\right\} - \min \left\{ t \,|\, Q(t) \ge 0.5 \, \max _t Q(t) \right\} \,. \end{aligned}$$

Tagged monocytes that are recruited later circulate for longer time periods. Increased migratory behaviour of foam cells leads to reduced circulation times. This is because higher values of $\sigma _e$ slow plaque growth, reducing the plaque size and the distance foam cells have to travel before exiting the plaque. As observed earlier in Fig. 5b and c, higher values of $\sigma _e$ also increase the velocity of foam cells near the medial boundary.

**Fig. 13:**
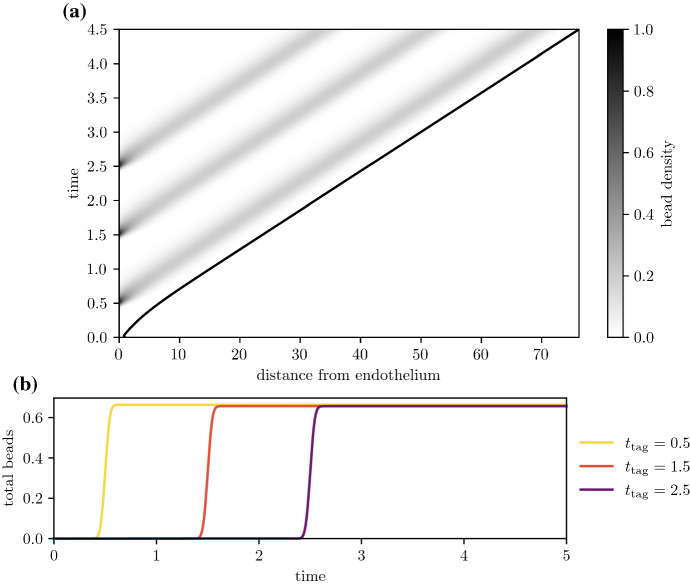
Bead circulation for different bead tagging times for a plaque with poor efferocytosis ($\mu _a = 40$, $\mu _e = 30$, $\sigma _e = 40$). Plot **a** shows the time evolution of the total bead distribution $q_f(x,t) + q_c(x,t)$, where beads are administered multiple times at $t_{\text {tag}} = 0.5,\, 1.5,$ and 2.5. Plot **b** compares the time evolution of the total bead quantity $Q(t) = \int _0^{R(t)} (q_f+q_c) \textrm{d}x$ for the three bead dosages in (a)

For plaques with poor efferocytic uptake rates that contain mostly dead cells, bead-tagged foam cells will die, and the beads will remain in the plaque indefinitely. Figure 13a tracks the evolution of the bead distribution for a plaque with nonzero emigration velocity ($\sigma _e = 40$) and poor efferocytosis ($\mu _a = 40$, $\mu _e = 80$, so $\frac{\mu _e}{\mu _a}=0.5$). Like the high efferocytosis case, bead-carrying cells are pushed deeper into the plaque as new material enters through the endothelium. Here, however, beads remain equidistant from the medial boundary as the plaque grows. Figure 13a tracks bead totals *Q*(*t*) with time and shows that bead numbers peak soon after the monocyte tagging time $t_{\text {tag}}$ and continue to remain high at longer times. This suggests that bead-carrying cells are failing to clear from the plaque, as no live foam cells are available to emigrate and allow material to leave the intima.

## Discussion

In this work, we have constructed a new model for early atherosclerotic plaque growth that includes the effects of cell death on plaque development. A key aspect of our approach is the use of a locally mass-conservative multiphase framework with a free boundary. This allows us to model growth of the intima due to the accumulation of new material and also to model cell movement and emigration in a manner that respects local mass conservation.

Our model finds that dead cells are most plentiful in the deep plaque, with a higher proportion of live macrophages closer to the endothelium. This is consistent with histological studies of early human aortic fatty streaks (Guyton and Klemp 1993), which contain macrophage-derived foam cells mostly in their middle and closer to the endothelium. Deposits of cholesterol crystals are observed deeper inside the intima, which are indicative of foam cell necrosis. Observations of more advanced plaques in humans and mice show a similar structure, where necrotic cores form deep in the plaque, and live macrophages tend to localise closer to the endothelium, albeit with a much larger necrotic core than in early fatty streaks (MacNeill et al 2004; Dickhout et al 2011).

Our model also suggests that advection is the primary mass transport mechanism that pushes material deeper into the plaque. The multiphase framework we use is highly idealised, where we include a simple mixture velocity advection term to help enforce the no-voids condition. This is in contrast to a force-balance equation approach or a full continuum mechanical approach based on porous flow theory or fluid mechanics, where advection-type terms will arise indirectly from other assumptions. Nevertheless, the mixture velocity advection term here plays a similar role to the advection term that arises in porous flow plaque models (Friedman 2015; Cilla et al 2015), which is typically induced by transmural pressure gradients across the endothelium or intima.

Despite the necrotic core being a key feature of advanced plaques, the role of dead cells in plaque development is largely neglected by other spatial models, and those spatial models that include cell death tend to model it via a simple unbalanced sink term. Ours is also the first spatially structured plaque model to explicitly consider efferocytosis, although Ford et al (2019) consider efferocytosis in a lipid-structured model without spatial structure. Fok (2012) presents a reaction-diffusion-chemotaxis model for necrotic core formation that explicitly includes dead cells. His model posits that the accumulation of macrophages deep in the plaque is driven mostly by chemotaxis toward extracellular lipid in the core, whereas in our model, advection is the primary means of mass transport into the deep plaque. Fok’s model also neglects the recycling of efferocytosed material by live cells, although it includes a term for the removal of dead cells by other efferocytosis-competent leukocytes.

In our model, the rate of efferocytic recycling relative to cell death is a key determinant of whether the deep plaque can support live foam cells. Higher rates of efferocytosis relative to death result in a higher proportion of live cells. These results are consistent with gene knockout studies in mice, which find that defective efferocytosis is correlated with large plaque size and higher numbers of dead cells (Kojima et al 2017). Crucially, our model undergoes a bifurcation as the ratio of the efferocytosis and death parameters changes, and complete foam cell death in the deep plaque is unavoidable if the ratio falls below a threshold value. Studies have explored the potential for using efferocytosis as a therapeutic target to treat cardiovascular disease and cancer (Kojima et al 2017), with strategies including disruption of the expression of “don’t-eat-me” signalling molecules (Kojima et al 2019), and the use of pro-efferocytic nanoparticles (Flores et al 2020) for example. Our model has the unfortunate implication that small improvements in macrophage efferocytic uptake may not be enough to induce regression of unstable advanced plaques. Larger improvements however may enable regression by increasing live foam cell counts.

Foam cell emigration in our model is critical in slowing plaque growth and can allow plaques to stabilise if emigration is large enough. This is consistent with experimental studies in mice in which the CCR7 receptor is blocked. CCR7 is involved in macrophage emigration, and blocking the receptor leads to larger plaques with impaired macrophage migration (Luchtefeld et al 2010). Intuitively, model plaques that are larger and plaques with slower foam cell emigration are characterised by increased retention of foam cells. This has implications for necrotic core formation, since foam cells that emigrate sooner are less likely to become necrotic. Emigration has been observed to enable steady states in other models, including the lipid-structured model by Ford et al (2019), and in ODE models by Cohen et al (2014) and Lui and Myerscough (2021). Emigration is typically neglected in existing spatial models however, and those that do exhibit steady states typically model emigration using unbalanced macrophage sink terms, rather than via emigration through the domain boundaries.

Inefficient efferocytosis and high rates of cell death inhibit foam cell emigration and plaque stabilisation by lowering the numbers of live foam cells. Below the threshold efferocytosis/death ratio required for the survival of deep plaque foam cells, emigration is effectively negligible. High rates of macrophage death and poor rates of emigration are both observed in plaques in hypercholesterolemic mice (Feig et al 2011). However, it remains unclear whether the lack of live cells specifically contributes to poor emigration rates in vivo, or whether both are a consequence of other factors such as excessive cholesterol loading. Attempts to block apoptotic pathways (using caspase inhibitors for instance) have been inconclusive due to the subsequent increase in necrosis in cells with dysfunctional apoptotic pathways (Martinet et al 2011).

Our model provides a good foundation for developing more detailed models of early plaque growth. One immediately relevant question is the effect of intracellular cholesterol, which is known to upregulate apoptotic pathways and disrupt normal cell function in macrophages (Tabas 2002). By separating the foam cell phase into two individual phases representing macrophages and intracellular cholesterol, we can model heterogeneity in foam cell cholesterol loads. The intracellular cholesterol phase would inherit the macrophages’ phase velocity, like the bead phase in this paper. This would allow us to incorporate some of the advantages of lipid-structured PDE models (Meunier and Muller 2019; Ford et al 2019; Watson et al 2022) into a spatial framework, such as cholesterol-dependent death and emigration rates.

Another important extension is the inclusion of HDL, which has multiple atheroprotective functions (Barter et al 2004; Brites et al 2017; Feig et al 2011), including the ability to accept cholesterol from foam cells, inhibit LDL oxidation, and stimulate macrophage emigration. Other modelling work has shown that increasing HDL levels can enable plaque stabilisation (Thon et al 2018b; Cohen et al 2014; Ford et al 2019; Friedman and Hao 2015). By extending our model to include HDL, we can explicitly consider how it reduces the accumulation of apoptotic and necrotic material.

The inclusion of a bead cargo species in our model also provides a promising means to explore further questions raised by Williams et al (2018) on macrophage motility and retention in plaques. Tracking individual cells is trivial in discrete cell-based models, but presents a challenge in a continuum modelling framework, and our bead model represents a novel method for tracking the movement of cells within a spatial PDE model. This approach may also prove useful in modelling other diseases where macrophage motility is an important question, such as in granulomas (Egen et al 2008) and tumour infiltration (Owen et al 2004).

We also remark on the ODE model formulated in Sect. 3.2. This submodel was obtained by approximating the full multiphase model as an advection-reaction equation and applying the method of characteristics. The ODE model provides a good approximation for deep plaque phase densities when the diffusive and chemotactic flux terms in (1) to (3) are much smaller than the bulk advection term, and the velocities of the various phases match closely. This breaks down near the endothelial boundary $x=0$, where the differing boundary fluxes of the various phases cause a phase velocity mismatch (Figs. 3 and 5). The same is observed at the medial boundary $x=R$ when the foam cell emigration flux is nonzero. Away from the boundaries, the characteristic ODE model provides a useful, analytically tractable tool with which to investigate dynamics caused by interphase interactions involving mass exchange. We study models of this form in more detail in future work. Perturbation analysis of the PDE about the ODE steady state may provide insight into the conditions necessary for the ODE steady state to be stable in the full multiphase system, and whether the system might be capable of exhibiting Turing-type instabilities and spatial patterning.

## Data Availability

Data sharing not applicable to this article as no data sets were generated or analysed during the current study.
